# Health-related quality of life and its determinants in patients with metastatic renal cell carcinoma

**DOI:** 10.1007/s11136-017-1704-4

**Published:** 2017-09-15

**Authors:** S. de Groot, W. K. Redekop, M. M. Versteegh, S. Sleijfer, E. Oosterwijk, L. A. L. M. Kiemeney, C. A. Uyl-de Groot

**Affiliations:** 1 Erasmus School of Health Policy and Management, Erasmus University Rotterdam, P. O. Box 1738, 3000 DR Rotterdam, The Netherlands; 2 Institute for Medical Technology Assessment, Erasmus University Rotterdam, P. O. Box 1738, 3000 DR Rotterdam, The Netherlands; 3 Department of Medical Oncology and Cancer Genomics Netherlands, Erasmus MC Cancer Institute, P. O. Box 5201, 3008 AE Rotterdam, The Netherlands; 4 Department of Urology, Radboud Institute for Molecular Life Sciences, Radboud University Medical Center, P. O. Box 9101, 6500 HB Nijmegen, The Netherlands; 5 Department for Health Evidence, Radboud Institute for Health Sciences, Radboud University Medical Center, P. O. Box 9101, 6500 HB Nijmegen, The Netherlands

**Keywords:** Health-related quality of life, EQ-5D, EORTC QLQ-C30, Cost-effectiveness analysis, Metastatic renal cell carcinoma, Targeted therapy

## Abstract

**Purpose:**

Based on improvements of progression-free survival (PFS), new agents for metastatic renal cell carcinoma (mRCC) have been approved. It is assumed that one of the benefits is a delay in health-related quality of life (HRQoL) deterioration as a result of a delay in progression of disease. However, little data are available supporting this relationship. This study aims to provide insight into the most important determinants of HRQoL (including progression of disease) of patients with mRCC.

**Methods:**

A patient registry (PERCEPTION) was created to evaluate treatment of patients with (m)RCC in the Netherlands. HRQoL was measured, using the EORTC QLQ-C30 and EQ-5D-5L, every 3 months in the first year of participation in the study, and every 6 months in the second year. Participation started as soon as possible following a diagnosis of (m)RCC. Random effects models were used to study associations between HRQoL and patient and disease characteristics, symptoms and treatment.

**Results:**

Eighty-seven patients with mRCC completed 304 questionnaires. The average EORTC QLQ-C30 global health status was 69 (SD, 19) before progression and 61 (SD, 22) after progression of disease. Similarly, the average EQ-5D utility was 0.75 (SD, 0.19) before progression and 0.66 (SD, 0.30) after progression of disease. The presence of fatigue, pain, dyspnoea, and the application of radiotherapy were associated with significantly lower EQ-5D utilities.

**Conclusions:**

Key drivers for reduced HRQoL in mRCC are disease symptoms. Since symptoms increase with progression of disease, targeted therapies that increase PFS are expected to postpone reductions in HRQoL in mRCC.

**Electronic supplementary material:**

The online version of this article (doi:10.1007/s11136-017-1704-4) contains supplementary material, which is available to authorized users.

## Introduction

Renal cell carcinoma (RCC) accounts for 90% of all kidney cancers [1]. While the prognosis of patients with localised disease treated with surgery is relatively good, the prognosis of patients with advanced or metastatic disease is poor. Median overall survival (OS) ranges from 7.8 months for patients with a poor risk to 43.2 months for patients with a favourable risk according to the Heng criteria [2]. Besides the impact of metastatic renal cell carcinoma (mRCC) on survival, mRCC can be associated with severe symptoms, such as cachexia and/or anorexia, asthenia and/or fatigue, pain, anaemia, and venous thromboembolism [3].

Since 2006, several new targeted therapies have been approved for the treatment of mRCC such as sunitinib, sorafenib, pazopanib and everolimus. In phase III studies, these therapies improved progression-free survival (PFS) of patients with mRCC over the diverse comparators [4–11], but the effect on OS was less pronounced, likely (partly) due to treatment crossover. It is assumed that one of the benefits of the new therapies is a delay in HRQoL deterioration as a result of a delay in progression of disease. Clinicians feel that a better PFS translates into a better HRQoL [12], but little data are available supporting this relationship. In the context of the high prices of targeted therapies which form a strain on health care budgets, it is important to establish whether indeed a delay in progression delays HRQoL deterioration.

This study is the first to provide insight into the most important determinants of HRQoL (including progression of disease) of patients with mRCC using data from a patient registry in the Netherlands [13]. Additionally, this study aims to assess if the association between progression and HRQoL, if one exists, is also captured by measures used in economic evaluations to assess benefit (i.e. EQ-5D).

## Patients and methods

### Study population

A patient registry (i.e. PERCEPTION) was created to evaluate treatment of patients with (m)RCC in the Netherlands. Patients with RCC (all stages) of any histological subtype diagnosed from 2011 until June 30th 2013 in 25 of 32 hospitals (both general and academic) in three regions in the Netherlands were invited to participate, and fill out HRQoL questionnaires. Eligible patients were identified through the hospitals’ registration systems. Additionally, the Netherlands Cancer Registry (NCR), which maintains a cancer registration database of all cancer patients in the Netherlands, was used to ensure that no patients were missed.

The research protocol was approved by the medical ethics committee of Radboud university medical center in Nijmegen (CMO Region Arnhem-Nijmegen) in May 2010. Informed consent was obtained from all patients participating in the HRQoL study.

### Data collection

Cancer-specific HRQoL was measured using the EORTC (European Organisation for Research and Treatment of Cancer) QLQ-C30 questionnaire (v3.0) [14]. This measure includes five functional scales (physical, role, emotional, social and cognitive), three symptom scales (fatigue, nausea & vomiting and pain), a global health status/QOL scale and six single items (dyspnoea, insomnia, appetite loss, constipation, diarrhoea and financial difficulties). In addition to the EORTC QLQ-C30, the EQ-5D-5L was used to measure HRQoL. The EQ-5D-5L is a preference-based generic measure, and measures HRQoL on five dimensions, i.e. mobility, self-care, usual activities, pain/discomfort and anxiety/depression. Each dimension includes five severity levels [15]. Patients were sent a HRQoL questionnaire every 3 months in the first year of participation in the study, and every 6 months in the second year. Participation started as soon as possible following a diagnosis of (m)RCC.

In addition to data on HRQoL, data on demographics, clinical and laboratory factors (to determine the patient’s risk group [16]) were collected retrospectively from individual patient records using uniform case report forms. Furthermore, data on treatment schemes and treatment endpoints (e.g. survival) were derived from patient records. Data collection stopped at the end of 2013.

### Statistical analyses

For each scale of the EORTC QLQ-C30, the average of the items that contributed to that scale was calculated. They were then linearly transformed in line with the EORTC QLQ-C30 scoring manual [17]. EQ-5D utilities were derived by combining the answers to the EQ-5D-5L with the Dutch EQ-5D- 5L tariff [18]. Mean EQ-5D utilities and HRQoL based on the EORTC QLQ-C30 were calculated by taking the average of the observations for each patient. The proportion of reported problems for each EQ-5D dimension were presented by taking the modus (i.e. the level reported most frequently) across observations for each patient. If two or more modes exist, the highest level was taken.

HRQoL was evaluated separately for the periods before and after progression of disease. In the period before progression of disease, a further distinction was made between wait-and-see and treatment with (first-line) targeted therapy. Treatment was assumed to last until progression of disease. Response including progression of disease was defined based on RECIST (as mentioned in the radiology report). As a substitute (if unavailable) data managers were instructed to register the response as indicated by the physician in the medical record. Patients who did not start therapy within the follow-up period were assumed to wait for therapy during the entire follow-up.

Since data on HRQoL were clustered, random effects models [19] were used to study associations between HRQoL (i.e. EORTC QLQ-C30 global health status and EQ-5D utility) and patient and disease characteristics, symptoms and treatment. Use of random effects models ensured that multiple measurements from the same patient were analysed appropriately and made it possible to distinguish between intraindividual and interindividual variation. Backward selection was used to select the covariates for the models; any non-significant covariates were excluded from the models one at a time (significance level of 0.20 for entering and 0.10 for removing the explanatory variables). To control for heteroscedasticity, random effects models with robust standard errors were estimated.

Additionally, random effects logit models [19] were used to study associations between the individual EQ-5D dimensions and patient and disease characteristics, symptoms and treatment. EQ-5D levels were dichotomised into ‘no problems/(i.e. level 1) and ‘problems’ (i.e. levels 2–5).

Missing data regarding patient and disease characteristics were handled using multiple imputations by chained equations. This method generated imputations based on a set of imputation models, one for each variable with missing values [20].

The significance level was set at *α* = 0.10. Data analyses were conducted using STATA statistical analysis software (StataCorp. 2013. Stata Statistical Software: Release 13. College Station, TX: StataCorp LP).

## Results

Four hundred eleven (m)RCC patients participating in the study completed 1630 questionnaires. The median number of questionnaires per patient was four (range 1–7). The number of questionnaires collected at each time point is provided in the Supplementary material (Fig. S1), as are the number of questionnaires per patient (Fig. S2). The average EORTC QLQ-C30 global health status of patients diagnosed with localised disease (336 patients, 1326 questionnaires) was 76 (SD, 15), and the average EQ-5D utility was 0.82 (SD, 0.17).

Eighty-seven patients had mRCC (i.e. metastatic disease at initial presentation or after an initial diagnosis with localised disease). Of these patients, eighty-two percent were male, and the median age at diagnosis was 63 years (Table 1). Twenty-six percent of the population did not receive any systemic therapy during follow-up. Of the patients receiving systemic therapy, the majority (80%) was treated with first-line sunitinib. Twenty-three patients also received a second-line therapy within the follow-up period; the majority of these patients was treated with everolimus (13/23). Thirty-one patients received radiotherapy during follow-up.

**Table 1:** Baseline characteristics at diagnosis

Variable	Patients (*n* = 87)
Male sex, *n* (%)	71 (82)
Age, median (range)	63 (40–79)
Non-clear cell pathology, *n* (%)	17 (20)
WHO performance status, *n* (%)	
0–1	82 (94)
2–4	5 (6)
More than one metastatic site, *n* (%)	48 (55)
Liver metastasis, *n* (%)	15 (17)
Lung metastasis, *n* (%)	48 (56)
Bone metastasis, *n* (%)	21 (24)
Brain metastasis, *n* (%)	3 (3)
Haemoglobin < LLN, *n* (%)	46 (52)
Neutrophil count > ULN, *n* (%)	18 (21)
Platelet count > ULN, *n* (%)	19 (22)
Corrected serum calcium > ULN, *n* (%)	26 (30)
Lactate dehydrogenase >1.5 times ULN, *n* (%)	11 (12)
Time since RCC diagnosis <1 year	78 (90)
MSKCC risk score, *n* (%)	
Favourable	6 (7)
Intermediate	54 (62)
Poor	27 (31)

*LLN* lower limit of normal, *ULN* upper limit of normal, *RCC* renal cell carcinoma, *MSKCC* Memorial Sloan Kettering Cancer Center

In total, 304 questionnaires were completed by patients with mRCC and the median number of questionnaires per patient was three (range 1–7).

Table 2 shows HRQoL during the different stages of the disease. The mean EORTC QLQ-C30 global health status was 67 (SD, 19). Patients primarily experienced problems with role functioning (i.e. doing daily activities and pursuing leisure time activities). Problems with emotional (i.e. feeling tense, irritable, depressed or worrying) and cognitive functioning (i.e. concentrating and remembering) were experienced less often. Symptoms most commonly reported were fatigue, pain, insomnia and dyspnoea. A statistically significant difference was found between the EORTC QLQ-C30 global health status before and after progression of disease, i.e. 69 (SD, 19) and 61 (SD, 22) (*p* = 0.022). All functioning scales significantly decreased, except for emotional and cognitive functioning. Two symptom scales significantly increased; patients reported more problems regarding dyspnoea (*p* = 0.031) and diarrhoea (*p* = 0.057) after progression than before progression of disease.

**Table 2:** Health-related quality of life based on the EQ-5D and QLQ-C30

	Total *n* = 87 patients (304 obs.)	Before progression *n* = 81 patients (246 obs.)			After progression *n* = 27 patients (58 obs.)
	Mean (SD)	Total mean (SD)	No systemic therapy *n* = 47 (125 obs.*) Mean (SD)	First-line therapy *n* = 50 (119 obs.) Mean (SD)	Total mean (SD)
EQ-5D					
Utility	0.74 (0.19)	0.75 (0.19)	0.76 (0.21)	0.76 (0.18)	0.66 (0.30)**
EORTC QLQ-C30					
Global health status	67 (19)	69 (19)	69 (22)	70 (17)	61 (22)***
Functioning scales					
Physical functioning	69 (23)	71 (23)	73 (22)	69 (23)	62 (29)
Role functioning	59 (28)	61 (29)	61 (30)	62 (29)	52 (33)
Emotional functioning	79 (16)	80 (18)	77 (19)	82 (19)	73 (19)
Cognitive functioning	80 (20)	80 (22)	81 (21)	79 (25)	76 (22)
Social functioning	76 (22)	78 (22)	77 (20)	78 (22)	67 (28)
Symptom scales					
Fatigue	41 (25)	39 (27)	36 (27)	41 (27)	48 (30)
Nausea and vomiting	12 (17)	13 (20)	8 (13)	17 (24)	10 (12)
Pain	29 (24)	27 (24)	24 (25)	29 (26)	34 (30)
Single items					
Dyspnoea	24 (24)	23 (24)	23 (25)	26 (28)	29 (34)
Sleeping	28 (26)	26 (27)	24 (27)	27 (30)	35 (31)
Appetite loss	19 (26)	18 (25)	15 (26)	21 (26)	22 (32)
Constipation	10 (17)	9 (17)	12 (24)	5 (10)	12 (21)
Diarrhoea	20 (26)	19 (27)	13 (27)	23 (28)	22 (26)
Financial difficulties	10 (18)	9 (18)	9 (21)	11 (19)	8 (21)

*Obs* observations

*Observations of patients who died within 90 days after being diagnosed with *m*RCC were excluded from this subgroup (*n* = 2), since these measurements would not contribute to the estimation of the HRQoL of a patient awaiting therapy

**Mean EQ-5D utility of these patients before progression of disease (*n* = 21) was 0.76 (0.23)

***Mean EORTC QLQ-C30 global health status of these patients before progression of disease (*n* = 21) was 69 (20)

In the period before progression of disease, a similar HRQoL was found for a period without therapy (i.e. wait-and-see) and a period with therapy; mean EORTC QLQ-C30 global health statuses were 69 (SD, 22) and 70 (SD, 17), respectively. However, in the period before progression of disease, patients experienced fewer problems with emotional functioning during a period with therapy compared to a period without therapy (*p* = 0.067). Additionally, patients reported fewer problems regarding constipation (*p* = 0.072), but more problems regarding diarrhoea during a period with therapy compared to a period without therapy (*p* = 0.005).

The average EQ-5D utility was 0.74 (SD, 0.19). As with the EORTC QLQ-C30 global health status, a significant difference was found in EQ-5D utility before progression of disease and after progression of disease; the average EQ-5D utility before progression of disease was 0.75 (SD, 0.19), whereas the average EQ-5D utility after progression of disease was 0.66 (SD, 0.30) (*p* = 0.032). In the period before progression of disease, no significant difference was found between a period without therapy (i.e. wait-and-see) and a period with therapy; mean utilities were 0.76 (SD, 0.21) and 0.76 (SD, 0.18), respectively. In the Supplementary material, Figs. S3 and S4 provide a summary of mean EORTC QLQ-C30 global health statuses and mean EQ-5D utilities by time.

**Fig. 1:**
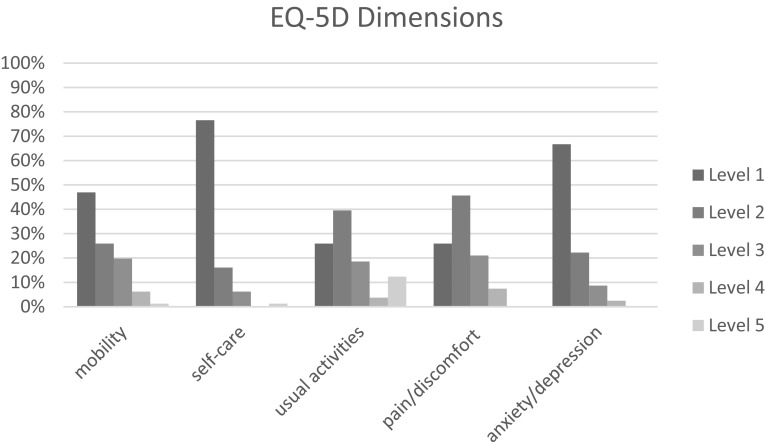
Proportion of patients reporting levels 1–5 by dimension, before progression of disease

**Fig. 2:**
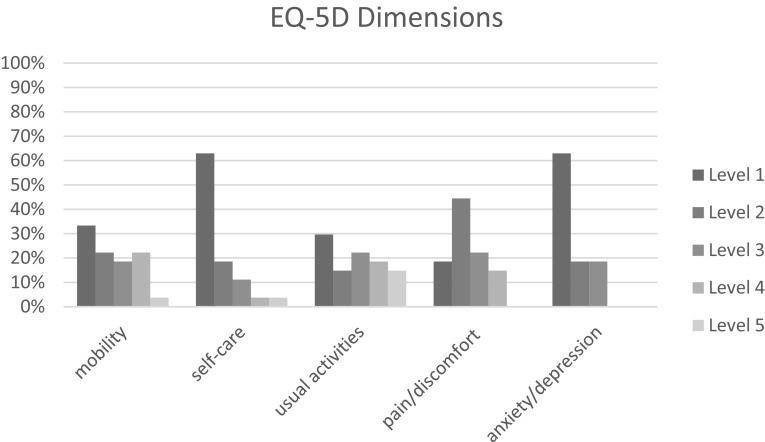
Proportion of patients reporting levels 1–5 by dimension, after progression of disease

Figures 1 and 2 show the proportions of patients reporting levels 1–5 by EQ-5D dimension, before progression of disease and after progression of disease. Both before and after progression of disease, most problems were reported on the mobility, usual activities and pain/discomfort dimensions.

Univariable analyses show several relationships between disease characteristics, symptoms and treatment, and HRQoL (Table 3). Patients with brain metastases and patients with progression of disease reported a lower HRQoL than the other patients. Patients with more than one metastatic site or bone metastases reported a lower EQ-5D utility, a relationship that was not seen in the EORTC QLQ-C30 global health status. Additionally, symptoms (i.e. fatigue, nausea and vomiting, pain, dyspnoea, insomnia, appetite loss, constipation and diarrhoea) were associated with a lower HRQoL. Lastly, patients treated with radiotherapy reported a worse HRQoL than patients not treated with radiotherapy.

**Table 3:** Associations between HRQoL and patient and disease characteristics, symptoms and treatment

	EQ-5D utility				EORTC QLQ-C30 global health status			
	Univariable analysis		Multivariable analysis		Univariable analysis		Multivariable analysis	
	Coefficient	SE	Coefficient	SE	Coefficient	SE	Coefficient	SE
Patient characteristics								
Male sex	0.077	0.069	NS		2.748	5.198	NS	
Age (per year)	−0.001	0.002	NS		−0.257	0.223	NS	
WHO performance score								
0–1								
2–4	−0.08	0.072	NS		−5.919	7.304	NS	
Disease characteristics								
More than one metastatic site	−0.068*	0.035	NS		−5.048	3.342	4.048*	2.276
Presence of liver metastases	−0.027	0.05	NS		−3.992	4.779	NS	
Presence of lung metastases	−0.021	0.041	NS		0.465	4.074	NS	
Presence of bone metastases	−0.085**	0.04	NS		−3.39	3.915	NS	
Presence of brain metastases	−0.285*	0.17	NS		−21.143*	10.239	−13.586***	2.438
MSKCC risk score								
Favourable								
Intermediate	0.015	0.062	NS		−0.431	8.924	NS	
Poor	0.054	0.063	NS		2.485	9.22	NS	
Progression of disease	−0.082**	0.036	NS		−6.897*	3	−3.859*	2.249
Disease duration (in months)	−0.002	0.001	NS		−0.081	0.117	NS	
Symptoms								
Fatigue	−0.004***	0.001	−0.003***	0.001	−0.451***	0.035	−0.316***	0.042
Nausea and vomiting	−0.001*	0.001	0.001**	0.001	−0.360***	0.05	NS	
Pain	−0.004***	0	−0.002***	0	−0.324***	0.036	−0.143***	0.035
Dyspnoea	−0.003***	0	−0.001***	0	−0.222***	0.04	NS	
Sleeping	−0.002***	0	NS		−0.219***	0.035	NS	
Appetite loss	−0.002***	0	NS		−0.274***	0.034	−0.111***	0.035
Constipation	−0.002***	0.001	NS		−0.186***	0.054	NS	
Diarrhoea	−0.001*	0	NS		−0.089**	0.04	NS	
Treatment								
Systemic therapy versus no systemic therapy	0.026	0.027	NS		−0.487	2.408	NS	
Radiotherapy	−0.150***	0.042	−0.115***	0.036	−10.017***	3.306	NS	
Model intercept			0.943***	0.016			85.380***	1.903
R^2^ (overall)				0.559				0.534
Wald test (p value)				<0.001				<0.001

Several comorbidities at diagnosis were considered for inclusion in the multivariable analyses, but all appeared to be not significantly associated with HRQoL

*SE* standard error, *NS* not significant

*Significant at *α* = 0.1

**Significant at *α* = 0.05

***Significant at *α* = 0.01

Multivariable analysis showed that the EORTC QLQ-C30 global health status decreased with the presence of fatigue, pain and appetite loss. Furthermore, the presence of brain metastases and progression of disease were associated with a worse EORTC QLQ-C30 global health status. A similar association was found between fatigue and pain, and the EQ-5D utility. Furthermore, EQ-5D utility scores decreased with the presence of dyspnoea and treatment with radiotherapy.

Although the univariable analyses showed several relationships between disease characteristics (e.g. the presence of bone or brain metastases and progression of disease) and HRQoL, these characteristics were no longer associated with a deterioration of HRQoL in multivariable analyses after correction for symptoms (at a significance level of 0.05 and 0.01, except for the presence of brain metastases in the model with the EORTC QLQ-C30 global health status as the dependent variable). This seems to imply that symptoms might increase due to progression of disease (and/or due to the spread of the cancer to the bone or brain), which explains the reduced HRQoL.

Table 4 shows that fatigue was associated with all EQ-5D dimensions, except with the mobility dimension; fatigue was associated with a greater frequency of problems regarding self-care, usual activities, pain/discomfort and anxiety/depression. Patients having pain reported problems with all EQ-5D dimensions more often, with the exception of anxiety/depression.

**Table 4:** Associations between the EQ-5D dimensions and patient and disease characteristics, symptoms and treatment

	Mobility		Self-care		Usual activities		Pain/discomfort		Anxiety/depression	
	OR	SE	OR	SE	OR	SE	OR	SE	OR	SE
Patient characteristics										
Male sex	0.149**	0.112	NS		0.095**	0.110	NS		NS	
Age (per year)	1.078**	0.032	NS		NS		NS		NS	
Disease characteristics										
Presence of liver metastases	4.427*	3.395	NS		NS		NS		NS	
Presence of lung metastases	NS		NS		NS		NS		0.300*	0.191
Presence of bone metastases	4.733**	2.961	NS		15.054***	14.768	NS		NS	
MSKCC risk score										
Favourable										
Intermediate	NS		NS		NS		0.041***	0.049	NS	
Poor	NS		NS		NS		0.143	0.176	NS	
Disease duration	NS		1.073**	0.033	NS		NS		NS	
Symptoms										
Fatigue	NS		1.044***	0.012	1.128***	0.028	1.034***	0.012	1.021**	0.010
Nausea and vomiting	0.967**	0.015	NS		NS		NS		NS	
Pain	1.029***	0.009	1.030***	0.010	1.029*	0.015	1.143***	0.023	NS	
Dyspnoea	1.025***	0.009	NS		1.024*	0.014	NS		NS	
Sleeping	NS		NS		NS		NS		1.016*	0.009
Appetite loss	1.031***	0.011	NS		NS		NS		NS	
Treatment										
Radiotherapy	NS		6.062***	3.971	NS		NS		NS	

Odds ratios based on models created using multivariable logistic regression

*OR* odds ratio, *SE* standard error

*Significant at *α* = 0.1

**Significant at *α* = 0.05

***Significant at *α* = 0.01

## Discussion

In this study differences were found between the health-related quality of life (HRQoL) of patients with metastatic renal cell carcinoma (mRCC) before and after progression of disease, with a reduced HRQoL after progression of disease. Progression of disease was no longer associated with a deterioration of HRQoL in multivariable analyses after correction for symptoms (at a significance level of 0.05 and 0.01). In line with Wilson and Cleary [21], a relationship between disease characteristics and symptoms was expected, which could explain why disease characteristics (such as progression) were no longer statistically significant in the multivariable analyses. Similarly, bone metastases were no longer associated with a deterioration of HRQoL in multivariable analyses. Since bone metastases can cause pain, then it is not surprising that bone metastases are not significantly associated with HRQoL once pain is included in the analysis. This seems to imply that symptoms increase due to progression of disease (and/or due to the spread of the cancer to the bone), which explains the reduced HRQoL.

Besides the relationship between symptoms and HRQoL, a significant association was found between radiotherapy and HRQoL (in the model with the EQ-5D utility as the dependent variable). It is possible that this observed association is not due to radiotherapy itself, but to the selection of which mRCC patients are to receive radiotherapy. That is, radiotherapy is mostly reserved for palliation of local and symptomatic disease or to prevent the progression of metastatic disease in critical sites (i.e. bones and brain) [22]. Either way, radiotherapy appears to be a significant determinant of HRQoL, even after correction for patient and disease characteristics (including bone and brain metastases) and symptoms.

The average EQ-5D utility of patients with mRCC was 0.74 compared to an average of 0.84 (SD, 0.18) in the Dutch population aged 60 to 69 [18]. Most patients (74%) in the study population were treated with a targeted therapy (the majority received sunitinib). The average EQ-5D utility of these patients was 0.76 before progression of disease. In a study by Cella et al., a similar EQ-5D utility was reported for patients treated with sunitinib (i.e. 0.75) [23]. In the economic evaluation of bevacizumab and sunitinib by Thompson-Coon and colleagues [24], a health state utility of 0.78 (95% CI 0.76–0.80) was used for progression-free survival and 0.70 (95% CI 0.66–0.74) for progressive disease. These utilities were derived from the data presented in the sunitinib submission to NICE and are somewhat higher than the utilities that we found in our study. The economic evaluation of sunitinib by Remák and colleagues [25] was based on the results of a phase II trial of sunitinib as second-line treatment in mRCC [26]; utilities of 0.72 and 0.76 were used for progression-free survival (i.e. during treatment or rest, respectively), whereas utilities of 0.63 and 0.55 were used for progressive disease (i.e. during second-line treatment or after termination of second-line treatment, respectively). The latter utilities are below the utilities found in our study, but this might be explained by differences in the study population, e.g. patients with progression on first-line cytokine therapy were enrolled in the phase II trial.

This study has several limitations that deserve mentioning. First, only 9% of the population (including those patients with RCC but not having metastatic disease) completed the 2-year follow-up period and filled in seven questionnaires. This is mainly because data collection stopped before many patients could be followed up for 2 years after diagnosis. That is, data collection stopped at the end of 2013, which meant that patients diagnosed after January 1st 2012 were not able to complete the full follow-up period. There are no reasons to expect important differences between the patients who did and did not complete the 2-year follow-up.

Second, a significant association between WHO performance status and HRQoL, and the MSKCC risk score and HRQoL was not found, although such a relationship would have been expected. The MSKCC risk score divides patients into three risk groups, and gives an indication of the life expectancy of patients with mRCC [16]. Whereas HRQoL was measured several times during the follow-up period, data on patient characteristics (e.g. WHO performance status) and disease characteristics (e.g. laboratory factors, which are part of the MSKCC risk score) were collected once before the start of each new treatment. As a consequence, too few observations on patient and disease characteristics might have been available to detect a significant association between WHO performance status and the MSKCC risk score, and HRQoL. Similarly, a significant association between comorbidities and HRQOL might have been expected, but data on comorbidities were only collected once (at diagnosis) which might explain why a significant association was not found. Nevertheless, the impact of comorbidities on HRQoL might be captured to some extent through age. Age appeared not to be significantly associated with HRQoL.

A third limitation is that our study sample was too small to find any difference in EQ-5D utilities between different types of targeted therapies, while these therapies differ in toxicity profiles [27]. Nevertheless, although adverse events have a high impact on HRQoL, an association between adverse events and HRQoL would not be found if the proportion of patients with grade 3 or 4 adverse events is relatively low. Hypertension and fatigue are the most commonly reported grade 3 or 4 adverse events in the randomised phase 3 trial of sunitinib [4], but these adverse events occurred in only 8 and 7% of the population. Therefore, a very large sample size is needed to find any difference in EQ-5D utilities between different types of targeted therapies. Additionally, it is unknown whether the improved HRQoL due to prolonged PFS outweighs reductions in HRQoL due to treatment-related adverse events. Importantly, this study did not find differences in HRQoL of patients treated with systemic therapy and patients not treated with systemic therapy, or between periods with or without systemic therapy. However, this study may have been underpowered to find such differences.

A fourth limitation is that no data were collected in the PERCEPTION-registry on assistance provided to patients who reported problems on one or more of the functioning scales of the EORTC QLQ-C30, while these patients could have received assistance to relieve their complaints. For example, patients could have received care at home to help with dressing and washing or emotional support by a psychologist or another healthcare professional. As a consequence, the impact of mRCC on HRQoL as presented in Table 2 might be underestimated.

Lastly, the total number of patients with mRCC was 233 in the 2011–2013 Cohort of the PERCEPTION-registry [13], while only 87 patients filled in one or more questionnaires about HRQoL. A comparison of the patient and disease characteristics and outcomes (in terms of overall survival) showed that the patients in the current study had a more favourable prognosis than the other patients in the PERCEPTION-registry. The impact on HRQoL as we estimated in this study is expected to be small, since we presented HRQoL associated with different stages of the disease.

To conclude, key drivers for reduced HRQoL in mRCC are symptoms of the disease. Since this study showed that symptoms increase with progression of disease, targeted therapies that increase PFS can help to delay loss in HRQoL. This study also showed that the EQ-5D is able to detect changes in HRQoL of patients with mRCC, as it found associations between well-known symptoms of mRCC and EQ-5D utilities. Similar associations were found between these symptoms and the disease-specific EORTC QLQ-C30.

## Electronic supplementary material

Below is the link to the electronic supplementary material.

Supplementary material 1 (PDF 213 KB)
