# Supplementary material for: Health-related quality of life and its determinants in patients with metastatic renal cell carcinoma
Source: Qual Life Res. 2017 Sep 15;27(1):115–24. doi: 10.1007/s11136-017-1704-4 (PMC5770482; doi:10.1007/s11136-017-1704-4)

Figure S1 shows the number of questionnaires collected at each time point. The number of questionnaires collected decreased with time. Figure S2 shows the number of questionnaires per patient.

Figure S1. Number of questionnaires at each time point

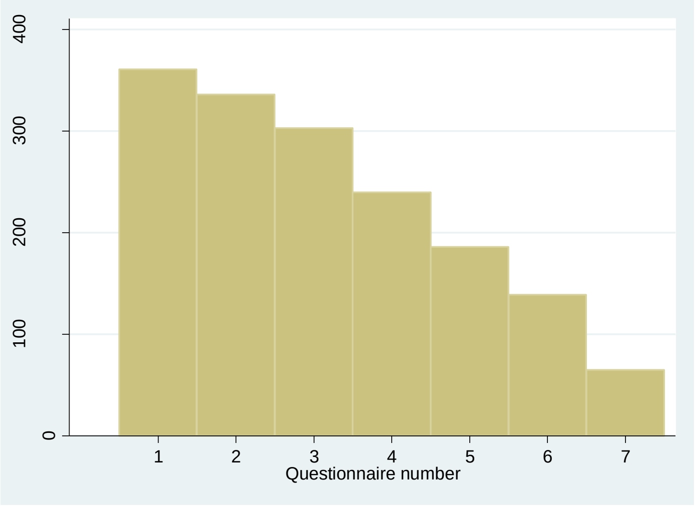

Figure S2. Number of questionnaires per patient

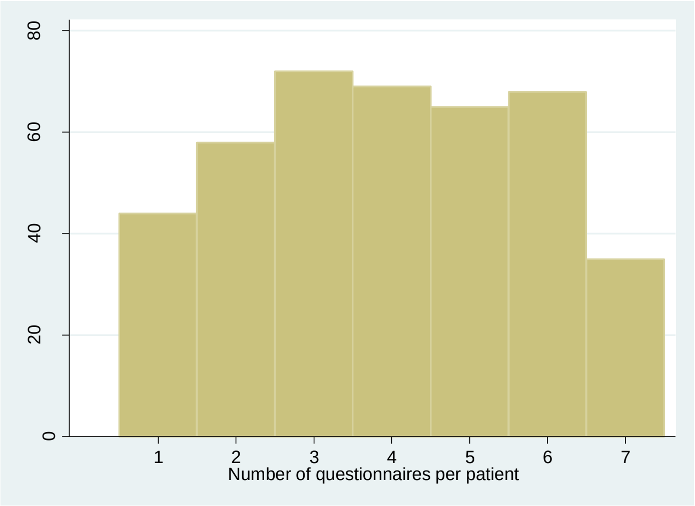

Figures S3 and S4 provide a summary of mean EORTC QLQ-C30 global health statuses and mean EQ-5D utilities by time (since diagnosis with metastatic disease).

Figure S3. Mean EQ-5D utilities by time

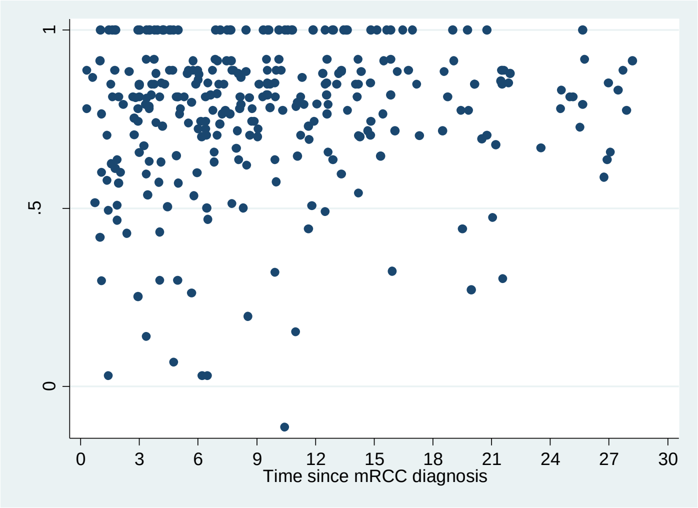

Figure S4. Mean EORTC QLQ-C30 global health statuses by time

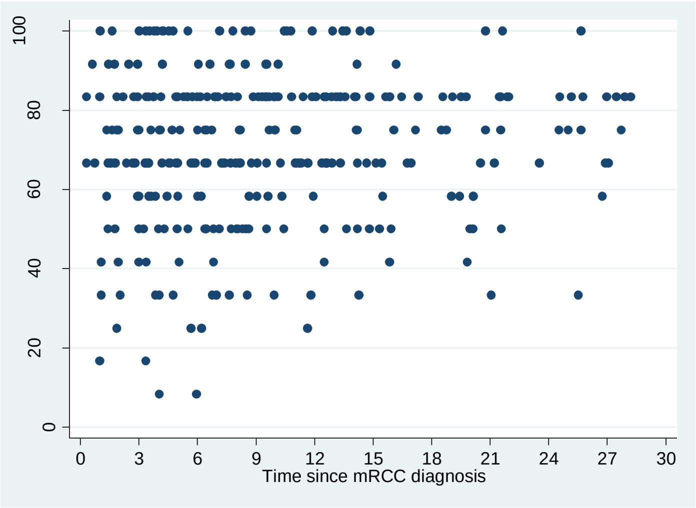

Supplement: Supplementary file 1 — Supplementary material 1 (PDF 213 KB) [file 11136_2017_1704_MOESM1_ESM.pdf]
